# Modelling of the Electric Field Distribution in Deep Transcranial Magnetic Stimulation in the Adolescence, in the Adulthood, and in the Old Age

**DOI:** 10.1155/2016/9039613

**Published:** 2016-03-16

**Authors:** Serena Fiocchi, Michela Longhi, Paolo Ravazzani, Yiftach Roth, Abraham Zangen, Marta Parazzini

**Affiliations:** ^1^CNR Consiglio Nazionale delle Ricerche, Istituto di Elettronica e di Ingegneria dell'Informazione e delle Telecomunicazioni IEIIT, Piazza Leonardo da Vinci 32, 20133 Milano, Italy; ^2^Department of Life Sciences, Ben-Gurion University of the Negev, 84105 Beersheba, Israel

## Abstract

In the last few years, deep transcranial magnetic stimulation (dTMS) has been used for the treatment of depressive disorders, which affect a broad category of people, from adolescents to aging people. To facilitate its clinical application, particular shapes of coils, including the so-called Hesed coils, were designed. Given their increasing demand and the lack of studies which accurately characterize their use, this paper aims to provide a picture of the distribution of the induced electric field in four realistic human models of different ages and gender. In detail, the electric field distributions were calculated by using numerical techniques in the brain structures potentially involved in the progression of the disease and were quantified in terms of both amplitude levels and focusing power of the distribution. The results highlight how the chosen Hesed coil (H7 coil) is able to induce the maxima levels of** E** mainly in the prefrontal cortex, particularly for the younger model. Moreover, growing levels of induced electric fields with age were found by going in deep in the brain, as well as a major capability to penetrate in the deepest brain structures with an electric field higher than 50%, 70%, and 90% of the peak found in the cortex.

## 1. Introduction

During the last decades, the prevalence of depression among a broad population *‎*[1] has led to increasing efforts to find new methods for alleviating the severe symptoms of that condition. Among them, transcranial magnetic stimulation (TMS) offers particularly appealing advantages, given mainly its noninvasive and painless use.

The efficacy of this technique is mediated by the electromagnetic induction of an electric field as described by Faraday's law. In brief, TMS is applied through short magnetic pulses delivered to the brain, produced by the passage of high electric current pulses in a coil placed over the patient's head *‎*[2].

The different shapes of the coil can modify the penetration depth and the focalization of the electric field and hence the likelihood of achieving specific targets in the brain. The aetiology of depression disorders is still not completely clear, but it is unlikely to affect only a single brain region or a neurotransmitter system. More probably, the heterogeneous symptoms of depressive disorders are driven by different brain structures, which interact in complex neurocircuits *‎*[3]. Most of the researches on depression attribute the progression of mood disorders to the impairment of areas belonging to limbic neurocircuits *‎*[4]. They comprehend the prefrontal cortex (PFC), the cingulate cortex, the hippocampus, some subcortical structures implicated in the reward circuit (such as the amygdala, the nucleus accumbens, and the ventral tegmental area) *‎*[5], and their connections with diencephalic areas (the hypothalamus, the thalamus, and the midbrain), just to name a few.

In order to target specifically these structures and in the meantime to overcome the undesirable side effects led by the conventional TMS coils *‎*[6–8], lately, new coils have been designed, with the aim of effectively stimulating deep brain targets with the so-called deep TMS (dTMS). This is the case of the family of the Hesed (H-) coils *‎*[9–11]: their current paths have a complex three-dimensional structure, which minimizes the nontangential coils elements thus allowing stimulating more efficaciously the structures that are further away from the scalp.

A particular type of H-coil, the H7 coil, recently used with positive outcomes in clinical trials *‎*[12, 13], is getting a prominent position among the other H-coils. The H7 coil is based on a new design that pledges a direct stimulation of frontal brain regions (i.e., the PFC) and it is specifically designed to reach deep brain regions without increasing the electric field levels in the more superficial cortical regions.

In order to link the role of the neural circuits mentioned above in the progressions of the depressive disorder with the efficacy of dTMS treatment with H-coils, a crucial role is played by the knowledge of the electric field distribution induced within the target tissues. In the meanwhile, the assessment of electric field (**E**) in different subjects could give peculiar information about a rationale and customized use of dTMS.

In this study we investigated, by a computational electromagnetic approach, the electric field induced by the H7 coil in the brain tissues involved in the depression disorders in four different human models of various ages and gender. With the specific aim of evaluating to which extent the age and the anatomical head morphology can affect the** E** distribution, we also evaluated its stimulation depth and focusing power over the four human models.

## 2. Materials and Methods

### 2.1. Human Models

Four high-resolution anatomical models of the virtual family *‎*[14] were used in this study: a child of 14 years of age (Louis), an adult female of 26 years of age (Ella), an adult male of 34 years of age (Duke), and an old male of 84 years of age (Glenn). Their different age allows considering and examining the potential application of dTMS by focusing on the main stages of body maturation and tissues diversity, that is, the adolescence, the adult age over both genders, and the old age. These human models have been developed by high-resolution segmentation of magnetic resonance (MR) images of healthy volunteers. They consist of up to 84 different tissues in the whole body and about 20 tissues at the brain level. Given the involvement in the pathogenesis of depression of some structures and subregions not distinguished within the human models used in this study, they were identified through a comparison with a brain atlas based on MR images. Specifically, in each human model, the left and the right amygdala, the ventral tegmental area (VTA), and the left and right nucleus accumbens have been included, all of them of particular interest for this study, together with the prefrontal cortex (PFC) and its subregions (i.e., the Dorsolateral Prefrontal Cortex, DLPFC, and the Medial Prefrontal Cortex, MPFC) and the anterior cingulate cortex. The dielectric properties of each tissue have been assigned according to literature data *‎*[15, 16] at the dTMS typical frequency.

### 2.2. Coil Configuration

In this study, we have modelled the H7 coil configuration. Briefly, it is formed by eight copper windings on each of the right and left wings, with a complete right-left symmetry in the coil configuration. In each wing, the eight windings are arranged on two layers of four windings each: the windings in the 2nd layer are placed on top of the windings on the 1st layer and adjacent to them. In each layer, the four windings are concentric ellipses, whose major axis ranges from 140 (outer ellipse) to 75 (inner ellipse) mm, and minor axis ranges from 125 to 70 mm. The wire diameter, including isolation material, is of 5.1 mm. The angle between the two planes on which the two groups of eight windings lie can be modified to conform to the shape and size of the head so that the coil windings are tangential to the head at the treatment position. The distance between the closest edges of adjacent couple of windings is 0.5 cm; the minimum distance between the right and left wings is of 3 cm.

**Figure 1:**
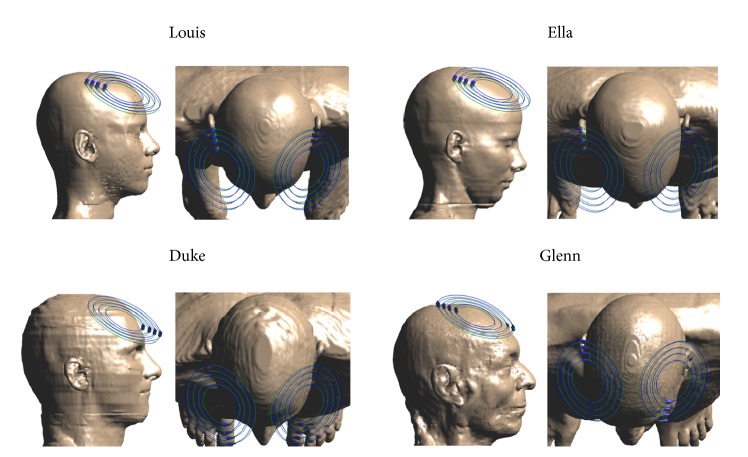
Sagittal and axial view of the four human models and relative position of the H7 coil. The green and blue ellipses indicate the four windings on the first and second layer for each wing.

Here the coil was placed in two different positions on specific anatomic sites of the head, according to the procedure commonly used in clinical practice. Firstly, it was placed over the leg motor cortex, to identify the minimum current able to generate in the cortical area of the motor cortex of tibialis anterior an electric field of 100 V/m. Then, the coil, fed with the same current, is moved 4 cm anteriorly and positioned over the frontal cortex (Figure 1).

The current delivered to the coil was a biphasic sinusoidal pulse at a frequency of 3 kHz.

### 2.3. Electric Field Numerical Simulations

The simulations were run using the simulation platform SEMCAD X (by SPEAG, http://www.speag.com/) *‎*[17], using the magneto quasi-static low frequency solver, which solves the Biot-Savart law and is based on the scalar potential finite element (SPFE) method. In the low frequency range, the dimensions of the computational domain are smaller than the free space wavelength and we can apply the magneto quasi-static approximation. Hence, the magnetic vector potential** A** is decoupled from** E**. Once** A** is calculated using the Biot-Savart law, electric field** E** can be determined by the scalar potential Φ, which is given by

(1) $$\begin{array}{c} -\nabla \cdot \sigma \nabla \Phi =j\omega \nabla \cdot \left(\;\sigma A\;\right) \end{array}$$

with *σ* as the tissue conductivity and *ω* as the angular frequency of the field.

The computational domain was truncated just below the shoulders level of the human models, where the level of the electric fields induced by the coil can be considered negligible. A uniform hexahedral meshing algorithm, made available by the software *‎*[17], with a maximum step of 1 mm, was chosen to allow the discretization of all tissues at the brain level, following an approach already used in dTMS literature [18]. It results in an applied mesh ranging from 57.5 to 117.3 million mesh cells.

### 2.4. Electric Field Characterization

The results will be presented in terms of both amplitude and spread of the electric field distribution. As to the amplitude, the maximum (named also “peak” in the following of this study) was taken as a reference for the amplitude distribution analysis. The maximum corresponds to the 99th percentile of the distribution in the pertinent tissue to avoid any possible computational instabilities *‎*[19, 20].

The focusing power of the** E** amplitude distributions inside different brain regions has been also estimated and presented in terms of the percentage of volume of the tissue that is exposed to** E** amplitude equal to or greater than 50%, 70%, 80%, and 90% of its peak in the cortex (V50, V70, V80, and V90, resp.). These parameters give a quantitative estimation of the capability to concentrate high** E** in a small volume; therefore the smaller these volumes are, the more the field is focused.

In order to compute the capability of the H7 coil to penetrate in the brain with a given amplitude strength, we assessed the maximum depth from the cortical surface of the deepest point with** E** amplitude equal to or higher than 50% (d50), 70% (d70), and 90% (d90) of the peak of** E** in the cortex. In detail, a brain centre and the direction of penetration along the line passing through that centre and the identified deepest point were determined. Then the depth was calculated by means of the distance between the point and its projection over the cortical surface, along the direction of penetration, following a method proposed by Guadagnin and colleagues *‎*[18]. A schematic representation of this method and the location of Cz, Nasion, and Inion of the 10-20 EEG system is shown in Figure 5 over Ella.

## 3. Results

**Figure 2:**
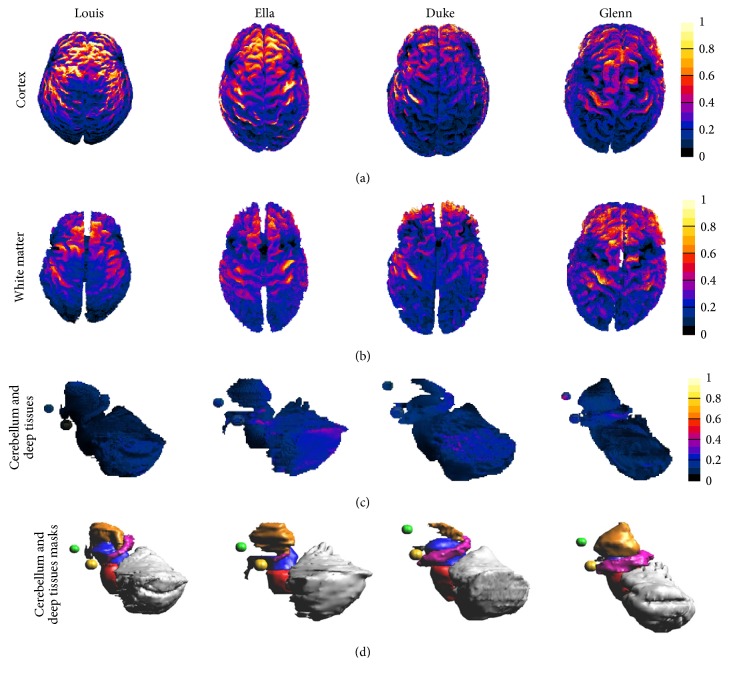
Surface amplitude distribution of** E** (V/m) induced by the H7 coil in the cortex and white matter (1st and 2nd rows) and in the cerebellum and deep brain tissues (3rd row) in Louis, Ella, Duke, and Glenn. For the sake of clarity, the 4th row shows the morphology of the deep brain tissues (the hippocampus in pink, the hypothalamus in black, the midbrain in blue, the thalamus in orange, the pons in red, the amygdala in yellow, and the nucleus accumbens in green) and the cerebellum (in grey). The amplitude field distributions of** E** are normalized with respect to the peak of** E** found in the cortex for each human model.

Figure 2 shows** E** amplitude spatial distribution induced by the H7 coil in different brain tissues for all the human models. All colour maps are normalized to the peak of** E** amplitude distribution in the cortex for each model. From top to bottom, the normalized amplitude distributions are shown on the cortex (top row), the white matter (second row), the cerebellum, and the deep brain tissues such as the hippocampus, the hypothalamus, the midbrain, the thalamus, the pons, the amygdala, the nucleus accumbens, and the VTA (third row).

Although** E** amplitude distributions in the cortex, in the white matter, and in the deeper tissues among the human models show different trends that depend on the individual head morphology, the general trend of the spatial distributions of the field amplitude shares some gross characteristics among the different human models. In particular, their pattern is qualitatively comparable, with a widespread distribution toward the prefrontal lobes and a dramatic decrease (of about more of the 75%) of the amplitude in the deepest tissues.

**Figure 3:**
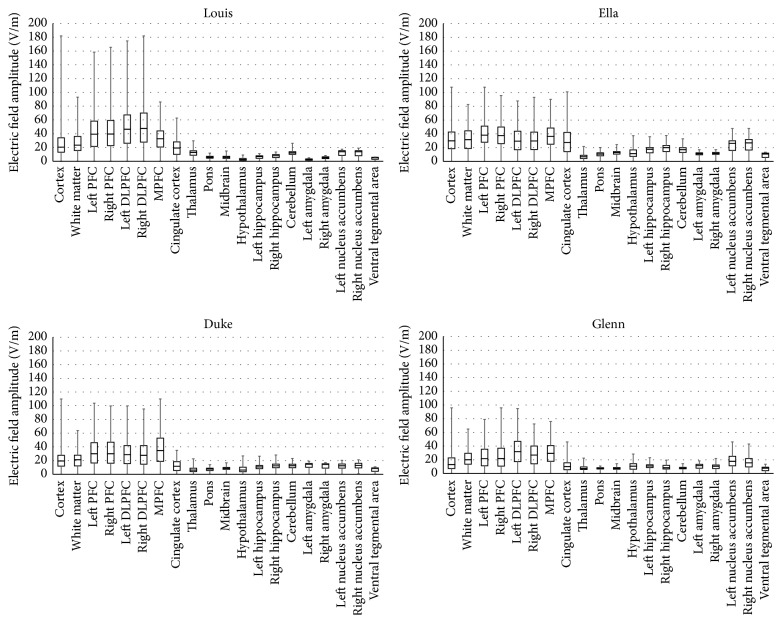
Descriptive statistic of** E** amplitude distributions (V/m) in different brain regions for all the four models.

Figure 3 shows the descriptive statistic of** E** amplitude distributions in different brain tissues for all the four models. The intramodel description indicates a consistent contraction of the peak values passing from the cortex to the white matter, with a decrease of 48.8%, 23.4%, 41.9%, and 32.2% for Louis, Ella, Duke, and Glenn, respectively, with respect to the cortex. These decreases are even more noticeable passing from the white matter to the deeper structures, where the percentages of reduction of the peak are 84.6%, 64.4%, 67.9%, and 64.5% for Louis, Ella, Duke, and Glenn, respectively, with respect to the white matter. Similar behaviours are shown also by the median values with a decrease of them from the white matter to the deeper structures of 66.5%, 51.9%, 50.4%, and 48.5% for Louis, Ella, Duke, and Glenn, respectively.

The location of the peaks varies across the various models: it occurs in the right DLPFC in Louis, in the left PFC in Ella, in the MPFC in Duke, and in the right PFC in Glenn. These data suggest a clear decreasing trend of the peak with the increasing of age, with a peak contraction ranging from about 40% for Duke and Ella to 47% for Glenn with respect to Louis. On the contrary, the comparison among the four panels of Figure 3 indicates an opposite trend in the deeper tissues, where the peak levels double passing from Louis to the adult models, in particular in the amygdala, the hippocampus, the hypothalamus, the nucleus accumbens, and the VTA.

To investigate better the characteristics of** E** amplitude distributions, Table 1 shows the V50 calculated over the cortex and its subregions and over the white matter. All the models show higher V50 in the DLPFC, with the exception of Duke for whom V50 is higher in the MPFC. Moreover, in the right DLPFC the V50 values are comparable in all the four models. A quite evident trend with age is shown in the left DLPFC, in which V50 ranges from 10.4% in Louis (the youngest model) to 20.1% in Glenn (the oldest model), and in the white matter, in which the V50 values almost quadruple from Louis to Glenn.

**Figure 4:**
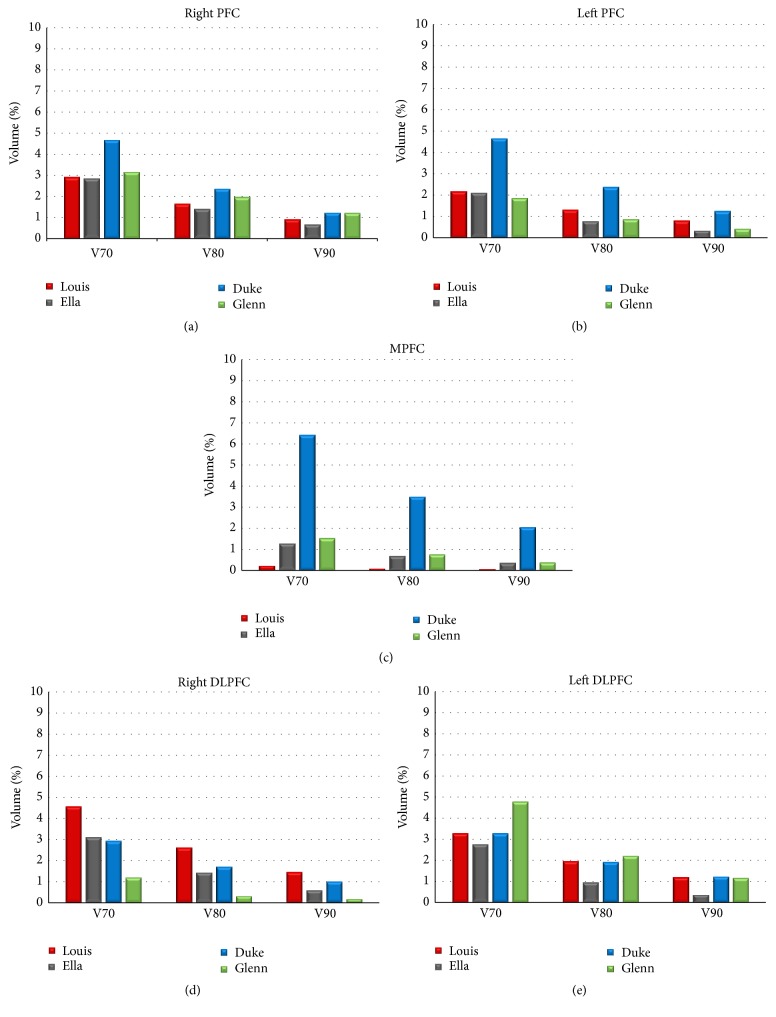
Percentage of the volume of the PFC (top panels), of the Medial Prefrontal Cortex (MPFC, panel in the middle), and of the Dorsolateral Prefrontal Cortex (DLPFC, bottom panels), showing an** E** amplitude higher than 70% (V70), 80% (V80), and 90% (V90) of the** E** peak in the cortex.

A more complete investigation of the focusing power is shown in Figure 4, as V70, V80, and V90 data estimated in the various brain regions of the prefrontal cortex taken into account in this study. The values found in the PFC and in the DLPFC are almost comparable among the models (with difference of 3% at maximum), whereas only for Duke the H7 coil is able to induce** E** amplitude higher than 90%, 80%, and 70% of its peak in the cortex in volumes of the MPFC much higher than in the other models.

**Figure 5:**
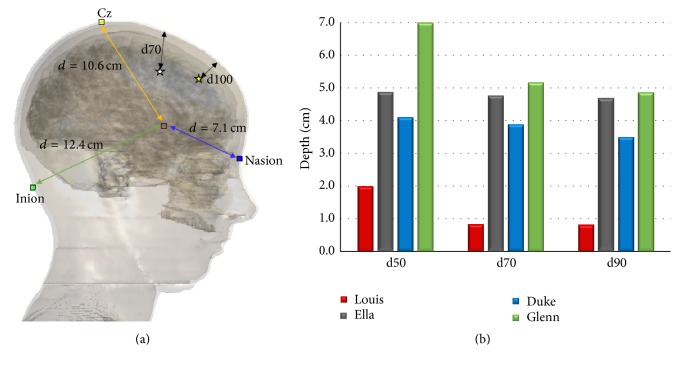
(a) Pictorial representation of the penetration depth calculation method, including the distance between the brain centre and the Cz, the Inion, and the Nasion of the 10-20 EEG system reference points for the Ella model (taken as an example). (b) Depth (cm) of the deepest point at 50%, at 70%, and at 100% of the peak of** E** from the cortical surface of the four models.

Figure 5 summarizes the penetration depth from the cortical surface of the deepest point with** E** amplitude equal to or higher than the 50% (d50), 70% (d70), and 90% (d90) of the peak of** E** in the cortex, along a direction toward the brain centre, for all human models (in the figure a pictorial description of the brain centre identification is also shown).

The capability to penetrate in the cortex is the highest for Glenn, reaching almost 7 cm with an intensity higher than the 50% of the peak and almost 5 cm with the 90% of the peak, whereas the lowest one is found for Louis, with a decrease with respect to Glenn's penetration depth of 3.5, 5.1, and 4.9 times for d50, d70, and d90, respectively. Moreover, it is higher in Ella than in Duke (with an average increase of 1 cm among the three distances), even if both models present a very slow decay (lower than the 10% passing from d50 to d90) among all the three depths of** E**.

## 4. Discussion and Conclusions

The consistent adoption of dTMS to treat symptoms of depressive disorders and the positive results attained by the clinical applications of that technique *‎*[21–27] have led to the introduction of new stimulating devices, specifically designed for inducing sufficient level of** E** in the deep brain structures involved in the progression of the disease. Among them, the H7 coil is a new-concept device, recently introduced in the clinical practice *‎*[12, 13]. This study aims at first assessing** E** amplitude distribution induced in these brain targets by that specific dTMS coil for the treatment of depressive disorder. Moreover, the effect of the morphological variability, even linked to age, has been also addressed. In view of the influence of the wide anatomical variability among subjects that could suffer from depressive symptoms,** E** distribution induced by the H7 coil has been compared indeed among subjects of different ages (from 14 to 84 years) and gender.

The results of this study are as such to indicate that the maxima levels of** E**, as expected, occur mainly in the prefrontal cortex, identified as the main cortical region critically involved in the depressive disorders and in other brain disorders such as obsessive-compulsive disorder (OCD), and close to the area over which the coils are placed (see Figures 2 and 3).** E** amplitude distributions show a high degree of variability among the subjects, with an evident influence of the dimension of the subject head, which helps to achieve the highest** E** amplitudes in the Louis head model, that is, the smallest one.** E** is also clearly influenced by the age of the modelled subjects: the lowest** E** levels have been found in Glenn, the oldest head model. This could be due to the cortical atrophy that affects elderly people *‎*[28] and should be strongly taken into account when dTMS is applied to the clinical treatment of neural diseases such as dementias.

The same behaviour can be found in the white matter (Figure 3), with the highest** E** peaks for Louis and the lowest for Duke and Glenn. Moreover, both the median and peak** E** levels in the cingulate cortex, belonging to the limbic lobe and strictly associated with the development of the obsessive-compulsive disorder, are highest in the female model (Ella), decrease in Louis, and are close to the levels of the deepest structure in the male and elderly models (Duke and Glenn).

These findings could be of overarching importance, since most of the connections of the neural circuits involved in the depression and in the OCD occur through the myelinated nerve cells projections of the white matter and through the cingulum.

In the deeper tissues, that is, in particular in the ones that are considered central for maintaining emotional stability and whose malfunction is linked to the pathophysiology of depression (i.e., the amygdala, the hippocampus, the hypothalamus, the nucleus accumbens, and the VTA),** E** is lower in the youngest model and shows comparable values in the adult models whereas it shows the highest value in Glenn. That implies a major decrease of** E** amplitude with the distance for the youngest model. These considerations are fully in line with the investigation of the penetration depth shown in Figure 5, in which Louis shows the lowest value of penetration depth and Glenn shows the highest one. That decrease could be due to the major thickness of the young prefrontal cortex (even 35% larger than that for adults and 50% with respect to elderly). On the contrary, the prefrontal atrophy, which characterizes the cortex in the elderly with the consequent increase of the CSF *‎*[29], could justify the high levels of** E** in the deepest tissues in Glenn.

The tissue localization of** E** peaks is different across the models (see Figure 3), whereas the focusing power (Figure 4) suggests a slight prevalence of the right side. This behaviour could be in line with some considerations about the therapeutic effects of dTMS, discussed in some studies *‎*[21, 30], which found laterality effect with a gain from excitatory dTMS over the left DLPFC and inhibitory dTMS over the right DLPFC. In any case, the H7 coil shows good capability to focus high levels of electric field in a very circumscribed volume, the focusing power being very high in all PFC areas (Figure 4), in particular in the youngest model. Lastly, the slighter major capability to penetrate into the deepest regions of Ella compared to Duke (Figure 5) could indicate some gender difference that can favour the application of these techniques in females, even though this should be better investigated in the future.

The variation of the levels and of the spread of the electric field levels across the models could have some important implications on the degree of neuromodulation within the brain tissues and, more specifically, on the excitability of the pyramidal cells around the target area. Indeed, since the size and the direction of the targeted cells and their axons are likely to vary across the model, this could affect the extent of the stimulation region [31].

However, since during their practices for brain targeting the clinicians are more interested in a general ranking of the brain regions where the field levels are higher instead of the minute details of current flow patterns, it could be sufficient to use some “reference human models” such as “adult” or “adolescent” and “elderly” in the planning of a dTMS treatment [32]. Our approach, therefore, could be an alternative to the use of individualized or customized models, to tune finely the dTMS treatment. Although this latter approach is in principle the optimal one, building a specific model for each patient can result in an extremely time-consuming and very expensive procedure.

In conclusion, this study shows that H7 coil is able to induce levels of electric field in the typical brain target areas for the treatment of depressive disorders comparable with the ones found for other H-family coils *‎*[33]. This conclusion is still valid also considering the effect of age. Indeed, the results presented here for Glenn and Duke are fully in line with the outcomes found for the same models by the H1 coil, that is, another coil of the H-family *‎*[33], which showed a slight decrease of the electric field levels in the prefrontal cortex and higher capability to reach deeper target in the brain for the elderly.

## Figures and Tables

**Table 1:** Percentage of the volume of different brain tissue where the amplitude of **E** is greater than 50% (V50) of the peak of **E** in the cortex, in each human model.

V50 (%)	Cortex	White matter	Left PFC	Right PFC	Left DLPFC	Right DLPFC	MPFC
Louis	12.9	1.0	7.1	8.3	10.4	12.8	0.7
Ella	5.3	1.7	10.7	11.3	12.9	11.9	5.9
Duke	4.7	2.7	17.9	17.9	12.2	11.1	24.5
Glenn	3.6	4.4	8.7	10.7	20.1	11.4	10.2
